# *trans*-Bromido(pyrimidinyl-κ*C* ^5^)bis­(triphenyl­phosphane-κ*P*)palladium(II)

**DOI:** 10.1107/S1600536811049348

**Published:** 2011-11-25

**Authors:** Hsiao-Fen Wang, Weng-Feng Zeng, Gene-Hsiang Lee, Kuang-Hway Yih

**Affiliations:** a Department of Hair Styling and Design, Hungkuang University, Shalu 433, Taichung, Taiwan; b Department of Applied Cosmetology, National Tainan Institute of Nursing, Tainan City 700, Taiwan; c Instrumentation Center, College of Science, National Taiwan University, Taipei 106, Taiwan; d Department of Applied Cosmetology, Hungkuang University, Shalu 433, Taichung, Taiwan

## Abstract

In the title complex, [PdBr(C_4_H_3_N_2_)(C_18_H_15_P)_2_], the geometry around the Pd atom is distorted square-planar with the Pd atom displaced by 0.0334 (14) Å from the BrP_2_C plane. The two Ph_3_P ligands are in *trans* positions, defining a P—Pd—P angle of 171.78 (5)°, while the pyrimidinyl and bromide ligands are *trans* to each other [C—Pd—Br = 174.63 (14)°].

## Related literature

For reactions in organic synthesis that form C—C bonds, see: Steffen *et al.* (2005 ▶); Beeby *et al.* (2004 ▶); Chin *et al.* (1988 ▶); Dobrzynski & Angelici (1975 ▶). For Pd—C(carbene) bond lengths, see: Cardin *et al.* (1972 ▶) and for Pd—Br bond lengths, see: Yih & Lee (2008 ▶); Yih *et al.* (2009 ▶). For related structures of pyrimidin­yl–metal complexes, see: Hong *et al.* (2002 ▶).
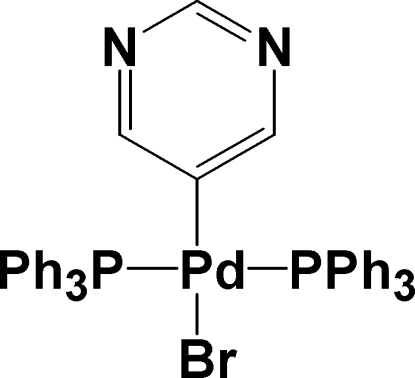


## Experimental

### 

#### Crystal data

[PdBr(C_4_H_3_N_2_)(C_18_H_15_P)_2_]
*M* *_r_* = 789.93
Monoclinic, 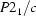

*a* = 15.0953 (10) Å
*b* = 12.0379 (8) Å
*c* = 19.8066 (13) Å
β = 109.7481 (13)°
*V* = 3387.5 (4) Å^3^
*Z* = 4
Mo *K*α radiation
μ = 1.85 mm^−1^
*T* = 150 K
0.35 × 0.20 × 0.12 mm

#### Data collection

Bruker SMART APEX CCD area-detector diffractometer
Absorption correction: multi-scan (*SADABS*; Bruker, 2001 ▶) *T*_min_ = 0.563, *T*_max_ = 0.808
19582 measured reflections
7764 independent reflections
5553 reflections with *I* > 2σ(*I*)
*R*_int_ = 0.079

#### Refinement

*R*[*F* ^2^ > 2σ(*F* ^2^)] = 0.052
*wR*(*F* ^2^) = 0.132
*S* = 1.01
7764 reflections
415 parameters
H-atom parameters constrained
Δρ_max_ = 0.87 e Å^−3^
Δρ_min_ = −0.98 e Å^−3^

### 

Data collection: *SMART* (Bruker, 2007 ▶); cell refinement: *SAINT* (Bruker, 2007 ▶); data reduction: *SAINT*; program(s) used to solve structure: *SHELXS97* (Sheldrick, 2008 ▶); program(s) used to refine structure: *SHELXL97* (Sheldrick, 2008 ▶); molecular graphics: *XP* in *SHELXTL* (Sheldrick, 2008 ▶); software used to prepare material for publication: *SHELXTL*.

## Supplementary Material

Crystal structure: contains datablock(s) I, global. DOI: 10.1107/S1600536811049348/bg2434sup1.cif

Structure factors: contains datablock(s) I. DOI: 10.1107/S1600536811049348/bg2434Isup2.hkl

Additional supplementary materials:  crystallographic information; 3D view; checkCIF report

## Figures and Tables

**Fig. 1.**
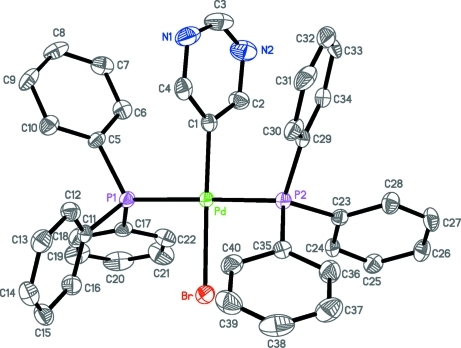
The molecular structure of (I), with 50% probability displacement ellipsoids.

## supplementary crystallographic information

### Comment

Palladium-complexes catalyzed formation of C—C bonds are some of the most important reactions in organic synthesis (Dobrzynski & Angelici, 1975). Intramolecular reductive elimination of Pd—N binuclear complex [Pd(µ-C_9_H_6_N)(µ-dppm)]_2_(Cl)_2_ yielding the organic compound 2,2'-biquinoline has been reported (Chin *et al.*, 1988). A pyridyl-bridged palladium complex was reported as an effective precatalyst for the Suzuki cross-coupling reactions of a variety of organoboronic acids and aryl bromides (Beeby, *et al.*, 2004). Pyrimidinyl nickel complexes have been used as catalysts for C—C coupling reactions (Steffen *et al.*, 2005). To our knowledge, no 3,5-pyrimidinyl palladium crystal structure has been described so far.

To synthesize the pyrimidinyl metal compound,  [Pd(PPh_3_)_4_] was used to react with 5-bromopyrimidine in dichloromethane at room temperature. As a result, a two triphenylphosphine displaced complex [Pd(Br)(C_4_H_3_N_2_)(PPh_3_)_2_] was isolated with 97% yield. The X-ray crystal structure analysis has been carried out to provide structural information.

The molecular structure of the title compound is shown in Fig. 1. The palladium atom has a distorted square planar environment, being displaced by 0.0396Å from the least-squares plane and with all angles about the Pd center within ± 2.6° of 90°. The Pd—C1 bond distance, 2.000 (5) Å, is longer than other Pd^II^-carbon(carbonyl) distances, and similar to Pd—C(carbene) distances (Cardin *et al.*, 1972). Two PPh_3_ ligands are in *trans* position: P1—Pd—P2, 171.78 (5)°, while the pyrimidinyl ligand and bromide are *trans* to each other: C1—Pd—Br, 174.63 (14)°. The phosphorus atoms approach tetrahedral geometry as expected. The largest angular deviation from ideal tetrahedral geometry is 119.31 (16)° for C5—P1—Pd. The mean Pd—N distance (> 4.25Å) indicates no bonding interaction between the nitrogen atom and the palladium metal atom. Within the pyrimidinyl ligand itself, the geometry is consistent with a significant partial double bond character in the C—C and C—N bonds. The C—N bond distances (1.315 (7) ~1.351 (6) Å) are typical for a C—N bond having partial double bond character and are certainly much shorter than the normal C—N (1.47 Å) single bond. The Pd—C1 (2.000 (5) Å), Pd—P (2.3175 (13), 2.3325 (13) Å) and Pd—Br (2.4907 (6) Å) lengths are in agreement with reported values (Yih *et al.*, 2008, 2009).

TThe ^31^P{^1^H} NMR spectra of (I) shows a singlet resonances at δ 24.2. In the ^1^H NMR spectra, the 2,6-H and 4-H protons of the pyrimidinyl group exhibit two singlet resonances at δ 7.75 and at δ 8.05. The ^13^C{^1^H} NMR spectra of (I) reveals two singlets at δ 151.9 and at δ 161.4 which are assigned to the 4-C and 2,6-C carbon atom of the pyrimidinyl group. There is a triplet resonance at δ 154.4 (^2^J_P—C_ = 5.91 Hz), which is assigned to the 1-C of pyrimidinyl group. In the FAB mass spectra, base peak with the typical Pd isotope distribution is in agreement with the [*M*^+^] molecular mass of (I).

### Experimental

The synthesis of the title compound (I) was carried out as follows. CH_2_Cl_2_ (20 ml) was added to a flask (100 ml) containing Pd(PPh_3_)_4_ (1.155 g, 1.0 mmol) and 5-Bromopyrimidine (0.191 g, 1.2 mmol) at ambient temperature. The mixture was stirred for about 1 day. The solvent was concentrated to 10 ml, and 20 ml of diethyl ether was added to the solution. The yellow solids were formed which were isolated by filtration (G4), washed with n-hexane (2 × 10 ml) and subsequently dried under vacuum yielding 0.774 g (97%) of [Pd(PPh_3_)_2_(C_4_H_3_N_2_)Br], (I). Spectroscopic data: ^31^P{^1^H} NMR: δ 24.2 (s, PPh_3_). ^1^H NMR: δ 7.27–7.70 (m, 30H, 2PPh_3_), 7.75 (s, 2H, 2,6-H of pyrimidinyl), 8.05 (s, 1H, 4-H of pyrimidinyl). ^13^C{^1^H} NMR: δ 128.3 (m, *o*-C of Ph), 130.2 (m, *p*-C of Ph), 134.5 (m, *m*-C of Ph), 151.9 (s, 4-C of pyrimidinyl), 154.4 (t, 1-C of pyrimidinyl, ^2^J_P—C_ = 5.91 Hz), 161.4 (s, 2,6-C of pyrimidinyl). MS (FAB, NBA, m/*z*): 789 [*M*^+^], 709 [*M*^+^ - Br], 630 [*M*^+^ - Br - pyrimidinyl]. Anal. Calcd. for C_40_H_33_BrN_2_P_2_Pd: C, 60.82; H, 4.21; N, 3.55. Found: C, 60.94; H, 4.31; N, 3.18.

### Refinement

H atoms were positioned geometrically and refined using a riding model, with C—H = 0.95Å and with *U*_iso_(H) = 1.2 times *U*_eq_(C).

### Crystal data

**Table:**

[PdBr(C_4_H_3_N_2_)(C_18_H_15_P)_2_]	*F*(000) = 1592
*M**_r_* = 789.93	*D*_x_ = 1.549 Mg m^−^^3^
Monoclinic, *P*2_1_/*c*	Mo *K*α radiation, λ = 0.71073 Å
Hall symbol: -P 2ybc	Cell parameters from 1845 reflections
*a* = 15.0953 (10) Å	θ = 2.7–21.4°
*b* = 12.0379 (8) Å	µ = 1.85 mm^−^^1^
*c* = 19.8066 (13) Å	*T* = 150 K
β = 109.7481 (13)°	Block, colourless
*V* = 3387.5 (4) Å^3^	0.35 × 0.20 × 0.12 mm
*Z* = 4	

### Data collection

**Table:**

Bruker SMART APEX CCD area-detector diffractometer	7764 independent reflections
Radiation source: fine-focus sealed tube	5553 reflections with *I* > 2σ(*I*)
graphite	*R*_int_ = 0.079
ω scans	θ_max_ = 27.5°, θ_min_ = 1.4°
Absorption correction: multi-scan (*SADABS*; Bruker, 2001)	*h* = −19→19
*T*_min_ = 0.563, *T*_max_ = 0.808	*k* = −15→11
19582 measured reflections	*l* = −25→25

### Refinement

**Table:**

Refinement on *F*^2^	Primary atom site location: structure-invariant direct methods
Least-squares matrix: full	Secondary atom site location: difference Fourier map
*R*[*F*^2^ > 2σ(*F*^2^)] = 0.052	Hydrogen site location: inferred from neighbouring sites
*wR*(*F*^2^) = 0.132	H-atom parameters constrained
*S* = 1.01	*w* = 1/[σ^2^(*F*_o_^2^) + (0.0532*P*)^2^] where *P* = (*F*_o_^2^ + 2*F*_c_^2^)/3
7764 reflections	(Δ/σ)_max_ < 0.001
415 parameters	Δρ_max_ = 0.87 e Å^−^^3^
0 restraints	Δρ_min_ = −0.98 e Å^−^^3^

### Special details

**Table:**

Geometry. All e.s.d.'s (except the e.s.d. in the dihedral angle between two l.s. planes) are estimated using the full covariance matrix. The cell e.s.d.'s are taken into account individually in the estimation of e.s.d.'s in distances, angles and torsion angles; correlations between e.s.d.'s in cell parameters are only used when they are defined by crystal symmetry. An approximate (isotropic) treatment of cell e.s.d.'s is used for estimating e.s.d.'s involving l.s. planes.
Refinement. Refinement of *F*^2^ against ALL reflections. The weighted *R*-factor *wR* and goodness of fit *S* are based on *F*^2^, conventional *R*-factors *R* are based on *F*, with *F* set to zero for negative *F*^2^. The threshold expression of *F*^2^ > σ(*F*^2^) is used only for calculating *R*-factors(gt) *etc*. and is not relevant to the choice of reflections for refinement. *R*-factors based on *F*^2^ are statistically about twice as large as those based on *F*, and *R*- factors based on ALL data will be even larger.

### Fractional atomic coordinates and isotropic or equivalent isotropic displacement parameters (Å^2^)

**Table:**

	*x*	*y*	*z*	*U*_iso_*/*U*_eq_	
Pd	0.22914 (2)	0.92402 (3)	0.419184 (18)	0.01789 (11)	
Br	0.12499 (4)	0.89245 (4)	0.29305 (3)	0.03315 (16)	
P1	0.20103 (9)	1.11231 (10)	0.39822 (6)	0.0196 (3)	
P2	0.23800 (8)	0.73438 (10)	0.44478 (6)	0.0193 (3)	
N1	0.3674 (4)	0.9979 (4)	0.6421 (2)	0.0379 (12)	
N2	0.4851 (3)	0.9595 (4)	0.5908 (3)	0.0399 (12)	
C1	0.3223 (3)	0.9531 (4)	0.5168 (2)	0.0203 (10)	
C2	0.4173 (3)	0.9424 (4)	0.5278 (3)	0.0278 (12)	
H2	0.4355	0.9215	0.4881	0.033*	
C3	0.4553 (4)	0.9840 (5)	0.6442 (3)	0.0414 (15)	
H3	0.5026	0.9928	0.6899	0.050*	
C4	0.3027 (4)	0.9829 (4)	0.5762 (3)	0.0267 (11)	
H4	0.2385	0.9941	0.5712	0.032*	
C5	0.2605 (3)	1.2118 (4)	0.4690 (2)	0.0223 (10)	
C6	0.3583 (3)	1.2133 (4)	0.4915 (3)	0.0260 (11)	
H6	0.3907	1.1635	0.4708	0.031*	
C7	0.4086 (4)	1.2873 (4)	0.5441 (3)	0.0307 (12)	
H7	0.4754	1.2876	0.5596	0.037*	
C8	0.3617 (4)	1.3609 (5)	0.5739 (3)	0.0326 (13)	
H8	0.3965	1.4115	0.6100	0.039*	
C9	0.2654 (4)	1.3611 (4)	0.5517 (3)	0.0316 (13)	
H9	0.2334	1.4118	0.5722	0.038*	
C10	0.2140 (4)	1.2861 (4)	0.4986 (3)	0.0277 (11)	
H10	0.1472	1.2864	0.4830	0.033*	
C11	0.0758 (3)	1.1278 (4)	0.3800 (2)	0.0203 (10)	
C12	0.0392 (3)	1.1108 (4)	0.4351 (3)	0.0257 (11)	
H12	0.0809	1.1015	0.4829	0.031*	
C13	−0.0575 (4)	1.1071 (4)	0.4209 (3)	0.0306 (12)	
H13	−0.0818	1.0973	0.4589	0.037*	
C14	−0.1174 (4)	1.1178 (4)	0.3517 (3)	0.0307 (12)	
H14	−0.1835	1.1135	0.3417	0.037*	
C15	−0.0831 (4)	1.1347 (4)	0.2967 (3)	0.0316 (12)	
H15	−0.1255	1.1432	0.2490	0.038*	
C16	0.0137 (4)	1.1395 (4)	0.3100 (3)	0.0282 (12)	
H16	0.0371	1.1507	0.2717	0.034*	
C17	0.2291 (3)	1.1726 (4)	0.3237 (2)	0.0220 (10)	
C18	0.1997 (4)	1.2801 (4)	0.3004 (3)	0.0343 (13)	
H18	0.1589	1.3190	0.3196	0.041*	
C19	0.2303 (5)	1.3305 (5)	0.2486 (3)	0.0438 (16)	
H19	0.2102	1.4036	0.2326	0.053*	
C20	0.2897 (4)	1.2739 (6)	0.2209 (3)	0.0441 (16)	
H20	0.3108	1.3083	0.1859	0.053*	
C21	0.3181 (4)	1.1688 (6)	0.2435 (3)	0.0430 (15)	
H21	0.3588	1.1304	0.2240	0.052*	
C22	0.2884 (4)	1.1175 (5)	0.2944 (3)	0.0310 (12)	
H22	0.3087	1.0441	0.3095	0.037*	
C23	0.3086 (3)	0.6555 (4)	0.4031 (3)	0.0200 (10)	
C24	0.3176 (3)	0.6939 (4)	0.3389 (3)	0.0234 (11)	
H24	0.2923	0.7641	0.3201	0.028*	
C25	0.3631 (3)	0.6297 (4)	0.3030 (3)	0.0272 (11)	
H25	0.3677	0.6554	0.2590	0.033*	
C26	0.4019 (3)	0.5289 (4)	0.3304 (3)	0.0284 (12)	
H26	0.4325	0.4850	0.3050	0.034*	
C27	0.3962 (4)	0.4918 (4)	0.3948 (3)	0.0300 (12)	
H27	0.4246	0.4233	0.4145	0.036*	
C28	0.3497 (4)	0.5535 (4)	0.4304 (3)	0.0274 (11)	
H28	0.3453	0.5267	0.4742	0.033*	
C29	0.2855 (3)	0.6946 (4)	0.5396 (2)	0.0212 (10)	
C30	0.2263 (4)	0.6664 (4)	0.5774 (3)	0.0276 (12)	
H30	0.1603	0.6619	0.5532	0.033*	
C31	0.2627 (4)	0.6449 (4)	0.6502 (3)	0.0345 (13)	
H31	0.2216	0.6261	0.6757	0.041*	
C32	0.3587 (5)	0.6506 (4)	0.6860 (3)	0.0387 (15)	
H32	0.3836	0.6347	0.7358	0.046*	
C33	0.4183 (4)	0.6795 (4)	0.6493 (3)	0.0361 (14)	
H33	0.4841	0.6849	0.6741	0.043*	
C34	0.3826 (4)	0.7007 (4)	0.5763 (3)	0.0271 (11)	
H34	0.4241	0.7193	0.5511	0.032*	
C35	0.1230 (3)	0.6683 (4)	0.4145 (2)	0.0210 (10)	
C36	0.1117 (4)	0.5551 (4)	0.4014 (3)	0.0320 (12)	
H36	0.1654	0.5104	0.4062	0.038*	
C37	0.0238 (4)	0.5062 (5)	0.3814 (3)	0.0405 (14)	
H37	0.0166	0.4287	0.3720	0.049*	
C38	−0.0542 (4)	0.5725 (6)	0.3752 (3)	0.0434 (16)	
H38	−0.1150	0.5399	0.3615	0.052*	
C39	−0.0443 (4)	0.6833 (5)	0.3887 (3)	0.0382 (14)	
H39	−0.0980	0.7273	0.3849	0.046*	
C40	0.0440 (3)	0.7326 (5)	0.4079 (3)	0.0295 (12)	
H40	0.0504	0.8103	0.4166	0.035*	

### Atomic displacement parameters (Å^2^)

**Table:**

	*U*^11^	*U*^22^	*U*^33^	*U*^12^	*U*^13^	*U*^23^
Pd	0.02109 (19)	0.01465 (19)	0.01659 (18)	0.00208 (14)	0.00463 (14)	0.00003 (15)
Br	0.0432 (3)	0.0249 (3)	0.0245 (3)	0.0058 (2)	0.0025 (2)	−0.0019 (2)
P1	0.0242 (6)	0.0159 (6)	0.0184 (6)	0.0005 (5)	0.0068 (5)	−0.0003 (5)
P2	0.0209 (6)	0.0172 (6)	0.0190 (6)	0.0009 (5)	0.0057 (5)	−0.0001 (5)
N1	0.051 (3)	0.038 (3)	0.023 (2)	−0.005 (2)	0.010 (2)	−0.006 (2)
N2	0.034 (3)	0.046 (3)	0.035 (3)	−0.009 (2)	0.005 (2)	0.002 (2)
C1	0.026 (3)	0.012 (2)	0.021 (2)	−0.0011 (19)	0.007 (2)	0.0037 (19)
C2	0.022 (3)	0.036 (3)	0.024 (3)	0.001 (2)	0.007 (2)	−0.001 (2)
C3	0.043 (4)	0.046 (4)	0.026 (3)	−0.015 (3)	−0.001 (3)	0.000 (3)
C4	0.027 (3)	0.016 (3)	0.038 (3)	−0.002 (2)	0.012 (2)	0.003 (2)
C5	0.034 (3)	0.016 (2)	0.017 (2)	−0.001 (2)	0.007 (2)	−0.001 (2)
C6	0.027 (3)	0.027 (3)	0.026 (3)	0.000 (2)	0.011 (2)	0.002 (2)
C7	0.031 (3)	0.029 (3)	0.028 (3)	−0.009 (2)	0.005 (2)	0.003 (2)
C8	0.046 (3)	0.031 (3)	0.022 (3)	−0.016 (3)	0.013 (3)	−0.008 (2)
C9	0.052 (4)	0.019 (3)	0.030 (3)	−0.005 (2)	0.022 (3)	−0.008 (2)
C10	0.034 (3)	0.025 (3)	0.026 (3)	−0.001 (2)	0.013 (2)	−0.001 (2)
C11	0.024 (3)	0.013 (2)	0.023 (2)	0.0009 (19)	0.007 (2)	−0.001 (2)
C12	0.028 (3)	0.022 (3)	0.025 (3)	0.000 (2)	0.006 (2)	0.003 (2)
C13	0.032 (3)	0.031 (3)	0.033 (3)	−0.001 (2)	0.017 (2)	−0.003 (2)
C14	0.025 (3)	0.027 (3)	0.040 (3)	0.003 (2)	0.010 (2)	0.006 (3)
C15	0.030 (3)	0.025 (3)	0.035 (3)	0.005 (2)	0.004 (2)	0.005 (2)
C16	0.032 (3)	0.029 (3)	0.024 (3)	0.004 (2)	0.011 (2)	0.003 (2)
C17	0.029 (3)	0.020 (3)	0.015 (2)	−0.003 (2)	0.005 (2)	0.001 (2)
C18	0.049 (4)	0.024 (3)	0.029 (3)	0.001 (3)	0.012 (3)	−0.001 (2)
C19	0.069 (4)	0.023 (3)	0.035 (3)	−0.013 (3)	0.012 (3)	0.007 (3)
C20	0.049 (4)	0.058 (4)	0.026 (3)	−0.026 (3)	0.013 (3)	0.003 (3)
C21	0.039 (3)	0.062 (5)	0.032 (3)	0.002 (3)	0.017 (3)	0.010 (3)
C22	0.027 (3)	0.038 (3)	0.025 (3)	0.000 (2)	0.006 (2)	0.004 (2)
C23	0.016 (2)	0.019 (2)	0.024 (2)	−0.0029 (19)	0.005 (2)	−0.004 (2)
C24	0.028 (3)	0.017 (2)	0.024 (2)	0.005 (2)	0.007 (2)	0.001 (2)
C25	0.032 (3)	0.026 (3)	0.026 (3)	−0.002 (2)	0.012 (2)	−0.001 (2)
C26	0.029 (3)	0.025 (3)	0.036 (3)	0.003 (2)	0.017 (2)	−0.005 (2)
C27	0.038 (3)	0.019 (3)	0.034 (3)	0.008 (2)	0.014 (2)	0.008 (2)
C28	0.034 (3)	0.021 (3)	0.030 (3)	0.001 (2)	0.014 (2)	0.001 (2)
C29	0.027 (3)	0.013 (2)	0.022 (2)	0.0030 (19)	0.005 (2)	−0.001 (2)
C30	0.042 (3)	0.018 (3)	0.026 (3)	−0.001 (2)	0.015 (2)	−0.002 (2)
C31	0.057 (4)	0.026 (3)	0.028 (3)	−0.002 (3)	0.024 (3)	−0.004 (2)
C32	0.068 (4)	0.022 (3)	0.021 (3)	0.013 (3)	0.009 (3)	0.003 (2)
C33	0.043 (3)	0.021 (3)	0.032 (3)	0.015 (2)	−0.005 (3)	0.003 (2)
C34	0.030 (3)	0.021 (3)	0.027 (3)	0.007 (2)	0.005 (2)	−0.003 (2)
C35	0.021 (2)	0.021 (3)	0.020 (2)	−0.0008 (19)	0.007 (2)	−0.001 (2)
C36	0.027 (3)	0.029 (3)	0.038 (3)	0.000 (2)	0.009 (2)	−0.004 (3)
C37	0.038 (3)	0.036 (3)	0.046 (4)	−0.014 (3)	0.012 (3)	−0.010 (3)
C38	0.030 (3)	0.064 (5)	0.036 (3)	−0.023 (3)	0.011 (3)	−0.008 (3)
C39	0.024 (3)	0.053 (4)	0.037 (3)	0.004 (3)	0.009 (3)	0.002 (3)
C40	0.031 (3)	0.033 (3)	0.024 (3)	0.000 (2)	0.010 (2)	−0.005 (2)

### Geometric parameters (Å, °)

**Table:**

Pd—C1	2.000 (5)	C18—C19	1.397 (8)
Pd—P1	2.3175 (13)	C18—H18	0.9500
Pd—P2	2.3325 (13)	C19—C20	1.379 (9)
Pd—Br	2.4907 (6)	C19—H19	0.9500
P1—C11	1.811 (5)	C20—C21	1.362 (8)
P1—C17	1.818 (5)	C20—H20	0.9500
P1—C5	1.832 (5)	C21—C22	1.379 (7)
P2—C35	1.817 (5)	C21—H21	0.9500
P2—C23	1.821 (5)	C22—H22	0.9500
P2—C29	1.834 (5)	C23—C28	1.399 (7)
N1—C3	1.324 (7)	C23—C24	1.401 (7)
N1—C4	1.351 (6)	C24—C25	1.379 (7)
N2—C3	1.315 (7)	C24—H24	0.9500
N2—C2	1.335 (7)	C25—C26	1.376 (7)
C1—C4	1.356 (7)	C25—H25	0.9500
C1—C2	1.381 (6)	C26—C27	1.382 (7)
C2—H2	0.9500	C26—H26	0.9500
C3—H3	0.9500	C27—C28	1.369 (7)
C4—H4	0.9500	C27—H27	0.9500
C5—C10	1.383 (7)	C28—H28	0.9500
C5—C6	1.391 (7)	C29—C30	1.388 (7)
C6—C7	1.385 (7)	C29—C34	1.401 (7)
C6—H6	0.9500	C30—C31	1.382 (7)
C7—C8	1.386 (7)	C30—H30	0.9500
C7—H7	0.9500	C31—C32	1.383 (8)
C8—C9	1.369 (7)	C31—H31	0.9500
C8—H8	0.9500	C32—C33	1.379 (8)
C9—C10	1.404 (7)	C32—H32	0.9500
C9—H9	0.9500	C33—C34	1.385 (7)
C10—H10	0.9500	C33—H33	0.9500
C11—C12	1.393 (7)	C34—H34	0.9500
C11—C16	1.394 (7)	C35—C36	1.386 (7)
C12—C13	1.392 (7)	C35—C40	1.391 (7)
C12—H12	0.9500	C36—C37	1.382 (7)
C13—C14	1.369 (7)	C36—H36	0.9500
C13—H13	0.9500	C37—C38	1.394 (8)
C14—C15	1.371 (7)	C37—H37	0.9500
C14—H14	0.9500	C38—C39	1.358 (8)
C15—C16	1.395 (7)	C38—H38	0.9500
C15—H15	0.9500	C39—C40	1.390 (7)
C16—H16	0.9500	C39—H39	0.9500
C17—C22	1.389 (7)	C40—H40	0.9500
C17—C18	1.396 (7)		
			
C1—Pd—P1	91.64 (13)	C18—C17—P1	120.2 (4)
C1—Pd—P2	89.49 (13)	C17—C18—C19	119.9 (5)
P1—Pd—P2	171.78 (5)	C17—C18—H18	120.0
C1—Pd—Br	174.63 (14)	C19—C18—H18	120.0
P1—Pd—Br	87.38 (3)	C20—C19—C18	119.9 (5)
P2—Pd—Br	92.24 (3)	C20—C19—H19	120.1
C11—P1—C17	108.2 (2)	C18—C19—H19	120.1
C11—P1—C5	107.1 (2)	C21—C20—C19	120.2 (5)
C17—P1—C5	99.9 (2)	C21—C20—H20	119.9
C11—P1—Pd	104.60 (15)	C19—C20—H20	119.9
C17—P1—Pd	117.19 (16)	C20—C21—C22	120.8 (6)
C5—P1—Pd	119.31 (16)	C20—C21—H21	119.6
C35—P2—C23	105.2 (2)	C22—C21—H21	119.6
C35—P2—C29	102.9 (2)	C21—C22—C17	120.4 (5)
C23—P2—C29	104.1 (2)	C21—C22—H22	119.8
C35—P2—Pd	112.25 (16)	C17—C22—H22	119.8
C23—P2—Pd	114.27 (16)	C28—C23—C24	118.3 (4)
C29—P2—Pd	116.86 (15)	C28—C23—P2	122.2 (4)
C3—N1—C4	113.8 (5)	C24—C23—P2	119.4 (4)
C3—N2—C2	115.1 (5)	C25—C24—C23	120.0 (5)
C4—C1—C2	114.0 (4)	C25—C24—H24	120.0
C4—C1—Pd	126.6 (4)	C23—C24—H24	120.0
C2—C1—Pd	119.3 (4)	C26—C25—C24	120.7 (5)
N2—C2—C1	124.0 (5)	C26—C25—H25	119.6
N2—C2—H2	118.0	C24—C25—H25	119.6
C1—C2—H2	118.0	C25—C26—C27	119.8 (5)
N2—C3—N1	127.9 (5)	C25—C26—H26	120.1
N2—C3—H3	116.1	C27—C26—H26	120.1
N1—C3—H3	116.1	C28—C27—C26	120.2 (5)
N1—C4—C1	125.1 (5)	C28—C27—H27	119.9
N1—C4—H4	117.5	C26—C27—H27	119.9
C1—C4—H4	117.5	C27—C28—C23	121.0 (5)
C10—C5—C6	119.4 (5)	C27—C28—H28	119.5
C10—C5—P1	124.0 (4)	C23—C28—H28	119.5
C6—C5—P1	116.5 (4)	C30—C29—C34	119.0 (5)
C7—C6—C5	120.1 (5)	C30—C29—P2	121.1 (4)
C7—C6—H6	119.9	C34—C29—P2	119.7 (4)
C5—C6—H6	119.9	C31—C30—C29	120.4 (5)
C6—C7—C8	120.2 (5)	C31—C30—H30	119.8
C6—C7—H7	119.9	C29—C30—H30	119.8
C8—C7—H7	119.9	C30—C31—C32	120.2 (5)
C9—C8—C7	120.3 (5)	C30—C31—H31	119.9
C9—C8—H8	119.9	C32—C31—H31	119.9
C7—C8—H8	119.9	C33—C32—C31	120.0 (5)
C8—C9—C10	119.9 (5)	C33—C32—H32	120.0
C8—C9—H9	120.1	C31—C32—H32	120.0
C10—C9—H9	120.1	C32—C33—C34	120.2 (5)
C5—C10—C9	120.1 (5)	C32—C33—H33	119.9
C5—C10—H10	119.9	C34—C33—H33	119.9
C9—C10—H10	119.9	C33—C34—C29	120.1 (5)
C12—C11—C16	118.9 (4)	C33—C34—H34	120.0
C12—C11—P1	119.5 (4)	C29—C34—H34	120.0
C16—C11—P1	120.9 (4)	C36—C35—C40	118.8 (5)
C13—C12—C11	120.8 (5)	C36—C35—P2	122.4 (4)
C13—C12—H12	119.6	C40—C35—P2	118.7 (4)
C11—C12—H12	119.6	C37—C36—C35	121.2 (5)
C14—C13—C12	119.5 (5)	C37—C36—H36	119.4
C14—C13—H13	120.2	C35—C36—H36	119.4
C12—C13—H13	120.2	C36—C37—C38	118.8 (6)
C13—C14—C15	120.7 (5)	C36—C37—H37	120.6
C13—C14—H14	119.7	C38—C37—H37	120.6
C15—C14—H14	119.7	C39—C38—C37	120.8 (5)
C14—C15—C16	120.6 (5)	C39—C38—H38	119.6
C14—C15—H15	119.7	C37—C38—H38	119.6
C16—C15—H15	119.7	C38—C39—C40	120.3 (5)
C11—C16—C15	119.6 (5)	C38—C39—H39	119.9
C11—C16—H16	120.2	C40—C39—H39	119.9
C15—C16—H16	120.2	C39—C40—C35	120.0 (5)
C22—C17—C18	118.8 (5)	C39—C40—H40	120.0
C22—C17—P1	120.6 (4)	C35—C40—H40	120.0
